# The prevalence rate, mortality, and 5-year overall survival of *Schistosoma japonicum* patients with human malignancy

**DOI:** 10.3389/fonc.2023.1288197

**Published:** 2023-12-06

**Authors:** Xue-Fei Liu, Shuai Ju, Ke-Ying Wang, Ying Li, Jin-Wei Qiang

**Affiliations:** ^1^ Department of Radiology, Jinshan Hospital, Fudan University, Shanghai, China; ^2^ Departments of Interventional Radiology, Jinshan Hospital, Fudan University, Shanghai, China

**Keywords:** *Schistosoma japonicum*, cancer, prevalence, mortality, five-year overall survival

## Abstract

**Background:**

Only a few studies have focused on the association between *Schistosoma japonicum* and human malignancy. The aim of this study was to update the prevalence rate, mortality, and 5-year overall survival of *S. japonicum* patients with human malignancy.

**Methods:**

From January 20, 2018, to January 31, 2021, 5,866 inpatients were included in the study. A total of 656 *S. japonicum* patients with malignancy were identified. Cases were stratified by gender and age groups. The cancer sites, prevalence rate, mortality, and 5-year overall survival of the patients were reported. The *S. japonicum* patients with malignancy were further divided into a non-digestive system tumor group (n = 309) and a digestive system tumor group (n = 347), including those with cancer in the esophagus, stomach, colon, rectum, liver, gallbladder, bile duct, or pancreas. Chi-squared test and odds ratio with confidence intervals were performed between these two groups.

**Results:**

Lung cancer was found the most common malignancy, accounting for 18.6% of all malignancies, followed by colorectal, stomach, liver, and gallbladder cancers. These five leading malignancies accounted for approximately 61.8% of all cases. Colorectal cancer was the leading cause of malignancy death, followed by lung, stomach, gallbladder, and liver cancers. These five leading causes of death accounted for approximately 55.6% of all death cases. Statistical significance was found in the prevalence rate between *S. japonicum* and non-*S. japonicum* patients with/without digestive system tumor (p < 0.001). The odds ratio of *S. japonicum* patients with digestive system tumors was 1.6 (95%CI: 1.4–1.9).

**Conclusion:**

*S. japonicum* contributes to a significant prevalence and mortality in digestive system tumors, including colorectal, stomach, liver, and gallbladder cancers.

## 1 Introduction

Human malignancy remains a major health problem, which has become one of the leading causes of death worldwide (1). It is estimated that over 20% of human malignancies are attributable to infections (2). Schistosomiasis is one of the most common parasitic infectious diseases worldwide. At least 254.1 million schistosomiasis-infected patients required preventive treatment in 2021 (https://www.who.int/news-room/fact-sheets/detail/schistosomiasis). A previous study reported that schistosomiasis infection had a potential relation to the occurrence of human malignancy (3).

There are three major *Schistosoma* species that infect humans: *Schistosoma mansoni*, *Schistosoma haematobium*, and *Schistosoma japonicum* (4). Among these, *S. haematobium* is reported as a definite carcinogen to humans (human carcinogens Group 1). *S. japonicum* is reported as a probable carcinogen to humans (human carcinogens Group 2). There is insufficient evidence for determining the carcinogenicity of *S. mansoni* in humans (human carcinogens Group 3) (3).

*S. japonicum* is endemic in Southeast Asia (primarily in mainland China). The cercariae of *Schistosoma* penetrate the skin of humans after the human host comes into contact with contaminated water. The schistosomula live within the veins of the human hosts and need approximately 5–7 weeks before becoming adults and producing eggs. The eggs remain in the body and would die within 1–2 weeks after being released by the female worm. However, the dead eggs can induce inflammation in the human host continually, which leads to inflammatory granulomatous and malignancies (4). Several lines of evidence indicate that *S. japonicum* is a causative agent in the development of liver cancer and colorectal cancer (5, 6). However, only a few studies have focused on the association between *S. japonicum* and human malignancy. Furthermore, the data linking *S. japonicum* to other malignancy sites except the liver and colon and rectum are insufficient. To the best of our knowledge, no study has investigated the prevalence rate, mortality, and 5-year overall survival of *S. japonicum* patients with human malignancy.

The aim of this study was to investigate the concomitance of *S. japonicum* and human malignancy. The prevalence rate, mortality, and 5-year overall survival of *S. japonicum* patients with malignancy were reported.

## 2 Materials and methods

### 2.1 Research ethics

This retrospective study was approved by the Ethics Committee of Jinshan Hospital, Fudan University (No. JIEC 2021-S55), and informed consent was waived. Patients’ confidentiality was protected. All the data were addressed anonymously. The personal information was appropriately de-identified.

### 2.2 Patients

**Figure 1:**
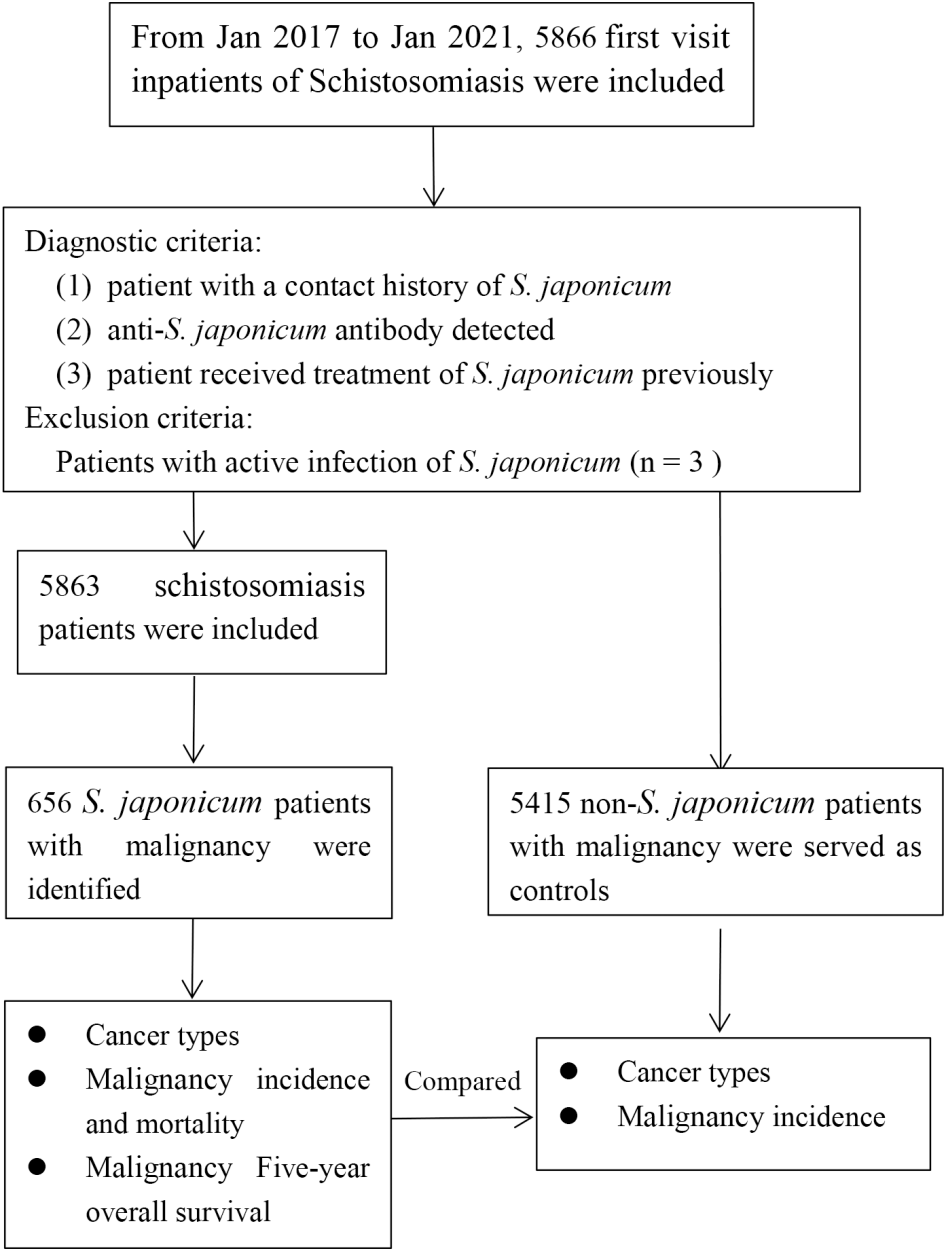
The workflow of this study.

From January 20, 2018, to January 31, 2021, 5,866 initial visit inpatients with schistosomiasis in our hospital were included in the study. All the patients were from an area (Jinshan District, Shanghai, China) that was highly endemic for *S. japonicum*. The diagnostic criteria were as follows: 1) patient with a contact history of *S. japonicum*; 2) anti-*S. japonicum* antibody detected by colloidal dye strip assay (DDIA), Schistosoma ovale test (COPT), enzyme-linked immunosorbent assay (ELISA), indirect red blood cell agglutination assay (IHA) or dot gold immunofiltration assay (DIGFA); and 3) patient received treatment of *S. japonicum* previously. The exclusion criteria were as follows: patients with positive results of rectal biopsy or stool examination were considered actively infected (n = 3). Finally, 5,863 patients were retained. The electronic medical records of all the patients were reviewed. Among these patients, malignancy cases diagnosed pathologically were identified. The malignancy sites were coded according to the International Statistical Classification of Diseases, 10th Revision (ICD-10). During the same time period, initial visit non-*S. japonicum* inpatients with pathologically diagnosed malignancy in our hospital were also enrolled. These patients served as the controls. According to the population structures of the *S. japonicum* patients, they were stratified into five groups by age (group 1, <50 years; group 2, 50–59 years; group 3, 60–69 years; group 4, 70–79 years; and group 5, ≥80 years). The workflow of this study is shown in **Figure 1**.

### 2.3 Clinical laboratory tests

The following were recorded: indicators reflecting liver function including serum alanine aminotransferase (ALT), aspartate aminotransferase (AST), albumin (ALB), total bilirubin (TTB), and prothrombin time (PT); indicators reflecting hematology including red blood cell (RBC), white blood cell (WBC), C-reactive protein (CRP), platelet count (PLT), and blood glucose (GLC); indicators reflecting renal function including urea (URE), creatinine (CRE), and uric acid (UAD); and indicators reflecting tumor markers including alpha-fetoprotein (AFP), carcino-embryonic antigen (CEA), cancer antigen 125 (CA125), and cancer antigen 199 (CA199).

### 2.4 The prevalence rate and mortality of *S. japonicum* patients with malignancy

For *S. japonicum* patients with malignancy, the prevalence rate (The number of cases of a certain tumor/The number of total tumor cases) and mortality (The number of death cases caused by a certain tumor/The number of total tumor cases) were calculated. Their gender- and age-specific prevalence rates and mortality were further calculated.

For non-*S. japonicum* patients with malignancy, the prevalence rate (The number of cases of a certain tumor/The number of total tumor cases) was calculated. Their gender- and age-specific prevalence rates were further calculated. The prevalence rates were compared between the *S. japonicum* and non-*S. japonicum* patients.

### 2.5 Gender- and age-specific 5-year overall survival of the *S. japonicum* patients with malignancy

For *S. japonicum* patients, the survival curves of each malignancy site were plotted. Their gender- and age-specific 5-year overall survival were also shown.

### 2.6 Statistical analysis

Statistical analysis was performed using R software (version 4.2.0; http://www.Rproject.org). The “epitools” package was used for the chi-squared test to compare the differences between categorical variables and to calculate the odds ratio with confidence intervals by unconditional maximum likelihood estimation and bootstrap method. The “survival” and “survminer” packages were used for survival analysis, which was assessed using the Kaplan–Meier curves *via* log-rank tests. p-Value less than 0.05 was considered statistically significant.

## 3 Results

### 3.1 Clinical characteristics of the patients

**Figure 2:**
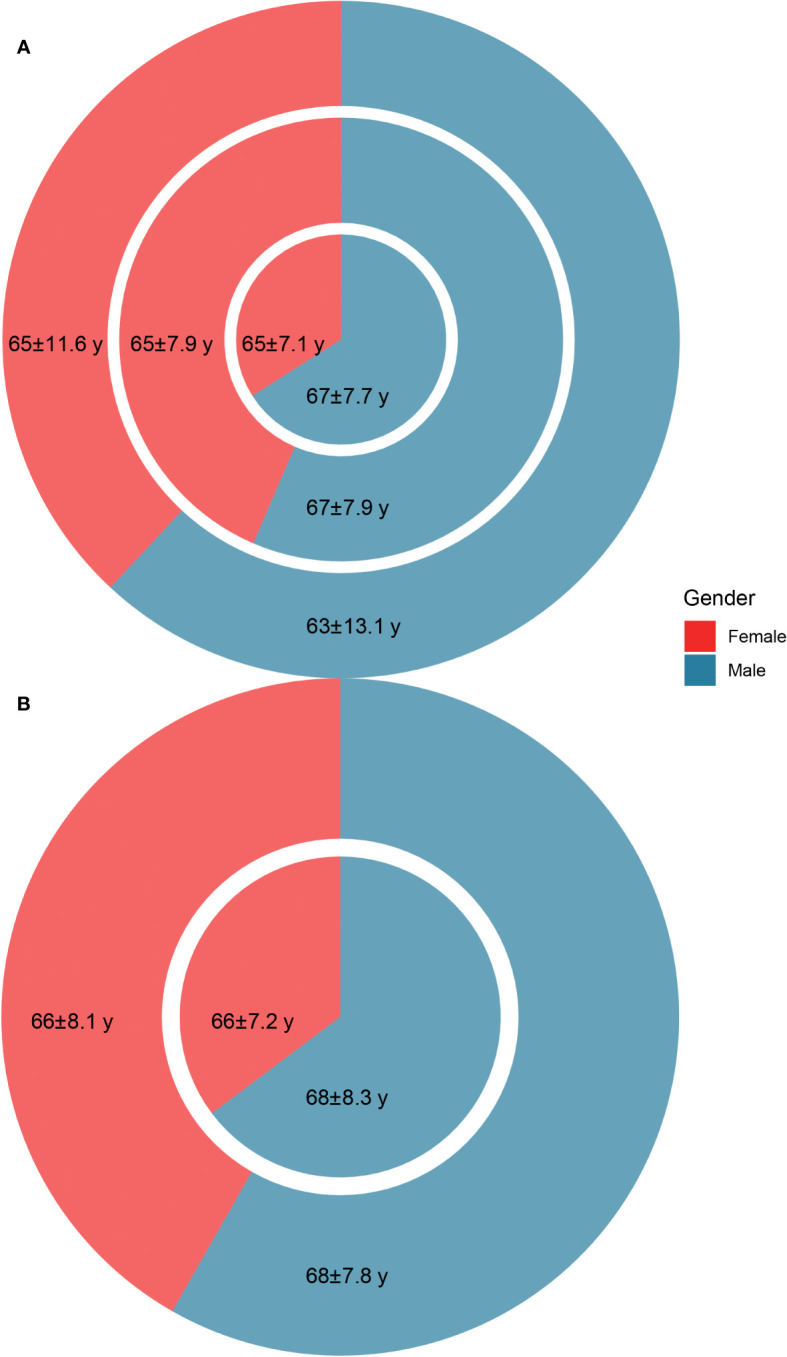
The age (mean ± standard deviation) and gender distribution of the patients. **(A)** The age and gender distribution of non-*Schistosoma japonicum* patients with malignancy (outer circle), *S. japonicum* patients without malignancy (middle circle), and *S. japonicum* patients with malignancy (inner pie). **(B)** The age and gender distribution of the total death cases of *S. japonicum* patients (outer circle) and the death cases of *S. japonicum* patients with malignancy (inner pie).

For *S. japonicum* patients, 5,863 initially admitted *S. japonicum* patients were identified, with 2,552 women and 3,311 men. In these patients, 656 newly diagnosed malignancy cases were identified, with 223 women and 433 men. For non-*S. japonicum* patients, 5,415 newly diagnosed malignancy cases were identified, with 2,062 women and 3,353 men (**Figure 2A**). For *S. japonicum* patients, a total of 661 death cases were identified, with 276 women and 385 men. Of these patients, 241 cases died from malignancy, with 85 women and 156 men (**Figure 2B**).

**Figure 3:**
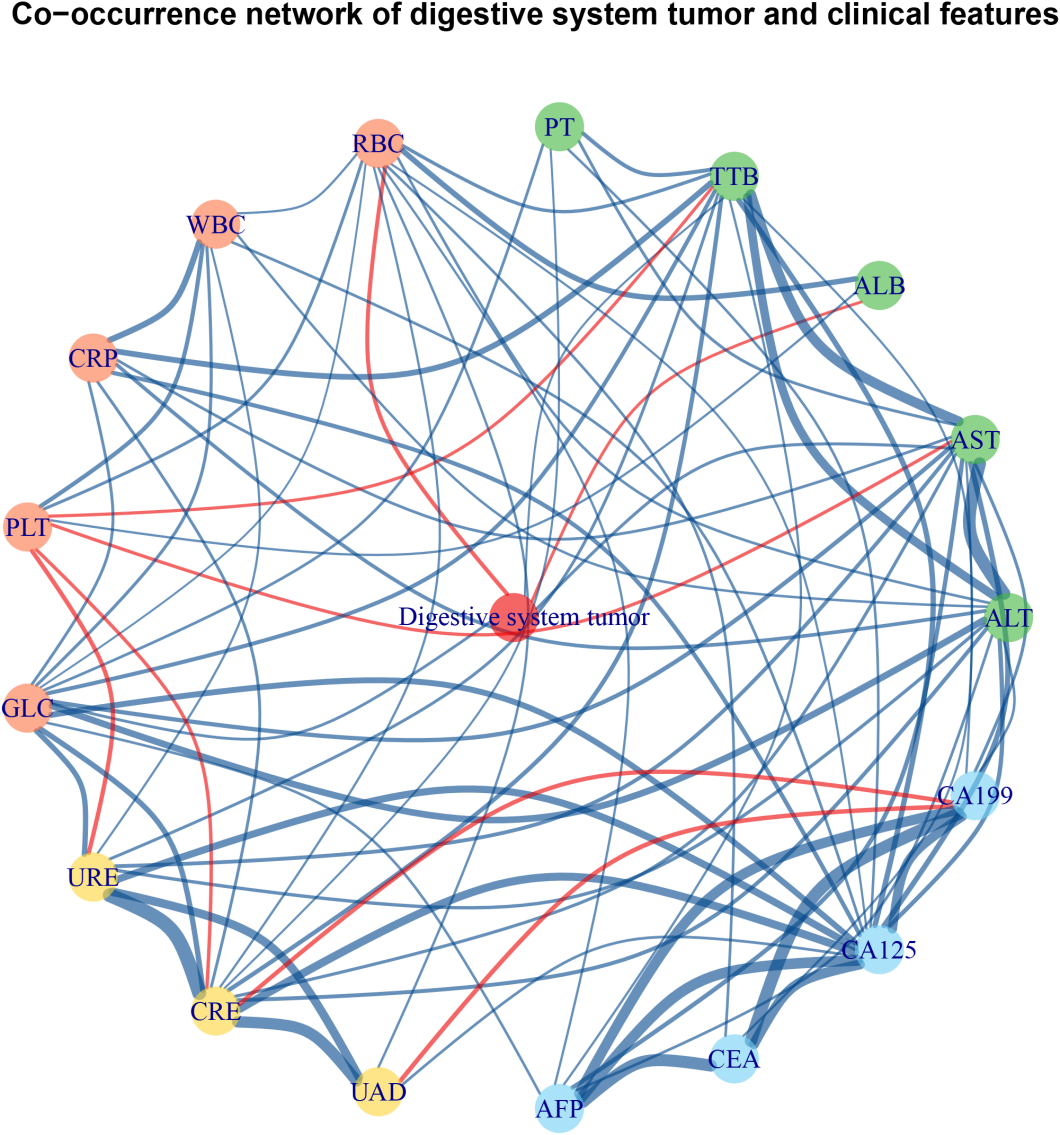
Co-occurrence network of digestive system tumor and clinical laboratory tests of *Schistosoma japonicum* patients with malignancy. The red edge indicates a negative correlation between two nodes, and the blue edge indicates positive correlation between two nodes. The green nodes are laboratory tests reflecting liver function. The red nodes are laboratory tests reflecting hematology. The yellow nodes are laboratory tests reflecting renal function. The blue nodes are laboratory tests reflecting tumor markers. ALB, albumin; ALT, alanine aminotransferase; AFP, alpha-fetoprotein; AST, aspartate aminotransferase; CA125, cancer antigen 125; CA199, cancer antigen 199; CEA, carcino-embryonic antigen; CRE, creatinine; CRP, C-reactive protein; GLC, blood glucose; PLT, platelet count; PT, prothrombin time; RBC, red blood cell; TTB, total bilirubin; UAD, uric acid; URE, urea; WBC, white blood cell.

The *S. japonicum* patients with malignancy were further divided into a non-digestive system tumor group (n = 309) and a digestive system tumor group (n = 347), including those with cancer in the esophagus, stomach, colon, rectum, liver, gallbladder, bile duct, or pancreas. The clinical characteristics of the two groups are shown in **Table 1**. Statistical significance was found in the prevalence rate between *S. japonicum* and non-*S. japonicum* patients with/without digestive system tumor (p < 0.001). The odds ratio of *S. japonicum* patients with digestive system tumors was 1.6 (95%CI: 1.4–1.9). The co-occurrence network of digestive system tumors and clinical laboratory tests is shown in **Figure 3**. The prevalence rate and mortality of *Schistosoma japonicum* and non-*S. japonicum* patients with malignancy are shown in **Table 2**.

**Table 1:** The clinical characteristics of *Schistosoma japonicum* patients with malignancy.

	Total (N = 656)	Digestive system tumor cases (N = 309)	Non-digestive system tumor cases (N = 347)	p
ALT	39.5 (58.6)	35.0 (51.8)	43.5 (63.9)	0.060
AST	49.7 (138)	43.8 (94.4)	54.9 (168)	0.294
ALB	51.7 (114)	57.4 (157)	46.7 (50.3)	0.254
TTB	31.5 (57.4)	21.7 (17.7)	40.4 (76.1)	<0.001
PT	19.9 (9.30)	19.3 (8.32)	20.5 (10.1)	0.081
RBC	5.1 (9.6)	6.1 (12.5)	4.3 (5.8)	0.023
WBC	7.6 (6.1)	8.0 (7.7)	7.2 (4.3)	0.110
CRP	33.8 (38.7)	33.5 (38.5)	34.0 (38.9)	0.866
PLT	180 (84.3)	183 (80.0)	177 (87.9)	0.372
GLC	7.3 (3.0)	7.1 (2.9)	7.6 (3.1)	0.043
URE	8.1 (12.4)	7.5 (4.2)	8.7 (16.6)	0.211
CRE	166 (752)	163 (742)	168 (761)	0.927
UAD	325 (177)	331 (127)	320 (212)	0.437
AFP	325 (2,640)	174 (162)	459 (3,620)	0.144
CEA	24.4 (65.5)	25.0 (54.0)	23.9 (74.3)	0.832
CA125	147 (301)	142 (287)	152 (312)	0.648
CA199	141 (631)	100 (115)	177 (860)	0.100

Data are presented as mean (SD).

ALB, albumin; ALT, alanine aminotransferase; AFP, alpha-fetoprotein; AST, aspartate aminotransferase; CA125, cancer antigen 125; CA199, cancer antigen 199; CEA, carcino-embryonic antigen; CRE, creatinine; CRP, C-reactive protein; GLC, blood glucose; PLT, platelet count; PT, prothrombin time; RBC, red blood cell; TTB, total bilirubin; UAD, uric acid; URE, urea; WBC, white blood cell.

**Table 2:** The prevalence rate and mortality of *Schistosoma japonicum* and non-*S. japonicum* patients with malignancy.

ICD-10	Position	*S. japonicum* patients			Non-*S. japonicum* patients	
		Mortality	Cases	Prevalence	Cases	Prevalence
A_O	All other sites	0.0%	1	0.2%	18	0.3%
C00–10, 12–14	Lip, oral cavity, and pharynx	0.0%	0	0.0%	55	0.9%
C11	Nasopharynx	0.0%	7	1.1%	94	1.6%
C15	Esophagus	6.8%	32	4.9%	274	4.7%
C16	Stomach	9.9%	70	10.7%	360	6.1%
C18	Colon	12.9%	87	13.3%	561	9.6%
C20	Rectum	3.8%	31	4.7%	317	5.4%
C22	Liver	9.1%	49	7.5%	266	4.5%
C23	Gallbladder	5.3%	29	4.4%	129	2.2%
C24	Bile duct	3.8%	17	2.6%	96	1.6%
C25	Pancreas	8.3%	32	4.9%	201	3.4%
C32	Larynx	0.8%	7	1.1%	73	1.3%
C33–C34	Lung	14.4%	122	18.6%	1,773	30.2%
C37, 38	Other thoracic organs	0.0%	0	0.0%	12	0.2%
C40, 41	Bone	0.0%	0	0.0%	6	0.1%
C43	Skin (melanoma)	0.8%	4	0.6%	6	0.1%
C44	Skin	0.0%	13	2.0%	18	0.3%
C45	Mesothelial cells	0.0%	1	0.2%	4	0.1%
C50	Breast	3.8%	15	2.3%	226	3.9%
C53	Cervix uteri	0.0%	1	0.2%	95	1.6%
C54	Corpus uteri	0.8%	2	0.3%	24	0.4%
C56	Ovary	0.0%	6	0.9%	38	0.7%
C61	Prostate	6.1%	42	6.4%	78	1.3%
C62	Testis	0.0%	0	0.0%	2	0.0%
C64	Kidney	1.5%	11	1.7%	302	5.2%
C66	Ureter	0.0%	2	0.3%	21	0.4%
C67	Bladder	1.5%	35	5.3%	173	3.0%
C71	Brain	0.0%	1	0.2%	26	0.4%
C73	Thyroid	0.8%	6	0.9%	34	0.6%
C82–C86, C96	Lymph nodes (lymphoma)	2.3%	15	2.3%	102	1.7%
C88	Blood (myeloma)	7.6%	18	2.7%	31	0.5%

### 3.2 Gender-specific prevalence rate of the patients with malignancy

**Figure 4:**
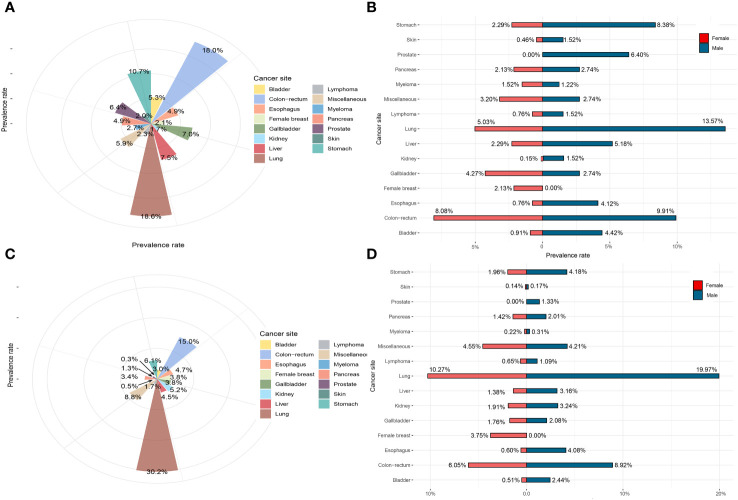
The prevalence rate of *Schistosoma japonicum* and non-*S. japonicum* patients with malignancy. **(A)** The prevalence rate of *S. japonicum* patients with malignancy (all the cases). **(B)** The prevalence rate of *S. japonicum* patients with malignancy (stratified by gender). **(C)** The prevalence rate of non-*S. japonicum* patients with malignancy (all the cases). **(D)** The prevalence rate of non-*S. japonicum* patients with malignancy (stratified by gender).

For *S. japonicum* patients, lung cancer was the most common malignancy, accounting for 18.6% of all cases, followed by colorectal cancer (18.0%), stomach cancer (10.7%), liver cancer (7.5%), and gallbladder cancer (7.0%). These five leading malignancies accounted for approximately 61.8% of all newly diagnosed malignancy cases. Lung cancer was found to be the most common malignancy in men, accounting for 13.6% of all cases, followed by colorectal cancer (9.9%), stomach cancer (8.4%), prostate cancer (6.4%), and liver cancer (5.2%). These five leading cancers accounted for approximately 43.5% of all newly diagnosed malignancy cases in men. For women, colorectal cancer was the most common malignancy, accounting for 8.1% of all cases, followed by lung cancer (5.0%), gallbladder cancer (4.3%), stomach cancer (2.3%), and liver cancer (2.3%). These five leading malignancies accounted for approximately 22.0% of all cases in women (**Figure 4**).

For non-*S. japonicum* patients, lung cancer was the most common malignancy, accounting for 30.2% of all cases, followed by colorectal cancer (15.0%), stomach cancer (6.1%), kidney cancer (5.2%), and esophageal cancer (4.7%). These five leading malignancies accounted for approximately 61.2% of all newly diagnosed malignancy cases. Lung cancer was found the most common malignancy in men, accounting for 20.0% of all cases, followed by colorectal cancer (8.9%), stomach cancer (4.2%), esophageal cancer (4.1%), and kidney cancer (3.2%). These five leading malignancies accounted for approximately 61.2% of all new cases in men. For women, lung cancer was the most common malignancy, accounting for 27% of all cancers, followed by colorectal cancer (6.1%), female breast cancer (3.8%), stomach cancer (2.0%), and kidney cancer (1.9%). These five leading malignancies accounted for approximately 40.8% of all cases in women (**Figure 4**).

### 3.3 Gender-specific mortality of the patients with malignancy

**Figure 5:**
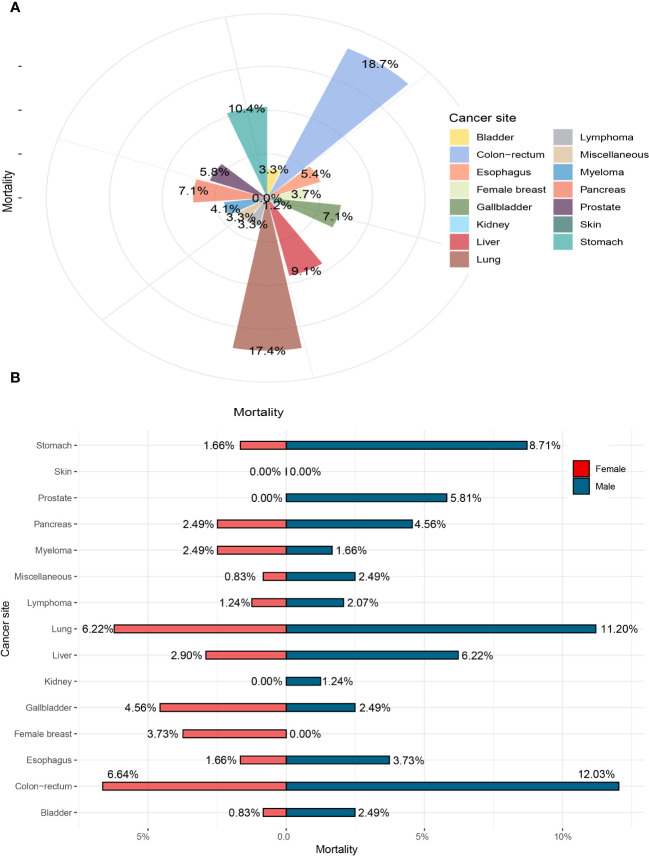
The mortality of *Schistosoma japonicum* patients with human malignancy. **(A)** The mortality of all the cases. **(B)** The mortality stratified by gender.

For *S. japonicum* patients, the mortality stratified by gender in all the cancer sites is shown in **Figure 5**. Colorectal cancer was the leading cause of malignancy death, which accounted for 18.7% of all death cases, followed by lung cancer (17.4%), stomach cancer (10.4%), liver cancer (9.1%), gallbladder cancer (7.1%), and pancreatic cancer (7.1%). These six leading malignancies accounted for approximately 55.6% of all death cases. Colorectal cancer was the leading cause of death cases in both genders. For men, colorectal cancer accounted for 12.0% of all death cases, followed by lung cancer (11.2%), stomach cancer (8.7%), liver cancer (6.2%), and prostate cancer (5.8%). The five leading malignancies accounted for approximately 43.9% of all death cases in men. For women, colorectal cancer accounted for 6.6% of all malignancy deaths, followed by lung cancer (6.2%), gallbladder cancer (4.6%), breast cancer (3.7%), and liver cancer (2.9%). These five leading malignancies accounted for approximately 24% of all death cases in women.

### 3.4 Age-specific prevalence rate and mortality of the patients with malignancy

For both *S. japonicum* and non-*S. japonicum* patients with malignancy, their prevalence rates increased with age (**Table 3**). The prevalence rates reached a peak in group 3 (60–69 years). The rates in group 4 (70–79 years) and group 2 (50–59 years) were comparable with those in group 3.

**Table 3:** The prevalence rate and mortality by age groups in *Schistosoma japonicum* and non-*S. japonicum* patients.

Gender	Cancer site	Schistosomiasis patients					Non-schistosomiasis patients				
		Group 1	Group 2	Group 3	Group 4	Group 5	Group 1	Group 2	Group 3	Group 4	Group 5
Prevalence rate	Bladder	0.0%	1.4%	2.4%	1.5%	0.0%	0.2%	0.4%	1.1%	1.0%	0.4%
	Colon and rectum	0.0%	3.7%	8.4%	5.6%	0.3%	2.0%	3.1%	4.7%	4.4%	2.1%
	Esophagus	0.2%	0.9%	2.3%	1.5%	0.0%	0.0%	0.6%	2.0%	1.7%	0.7%
	Female breast	0.0%	0.9%	0.8%	0.3%	0.2%	1.0%	1.3%	1.3%	0.4%	0.1%
	Gallbladder	0.0%	1.1%	4.0%	1.7%	0.3%	0.4%	0.8%	1.4%	1.0%	0.6%
	Kidney	0.2%	0.3%	0.8%	0.5%	0.0%	1.1%	1.4%	1.5%	1.0%	0.5%
	Liver	0.2%	0.9%	3.2%	3.0%	0.2%	1.0%	1.1%	1.2%	1.0%	0.7%
	Lung	0.5%	4.9%	10.2%	2.9%	0.2%	2.3%	6.0%	12.1%	9.3%	3.0%
	Lymphoma	0.0%	0.3%	1.7%	0.3%	0.0%	0.3%	0.4%	0.6%	0.4%	0.2%
	Miscellaneous	0.0%	1.8%	3.4%	0.8%	0.0%	2.3%	2.7%	2.3%	1.6%	0.5%
	Myeloma	0.0%	0.8%	1.2%	0.6%	0.2%	0.0%	0.1%	0.2%	0.3%	0.0%
	Pancreas	0.0%	1.1%	1.4%	2.3%	0.2%	0.2%	0.7%	1.4%	0.8%	0.6%
	Prostate	0.0%	0.8%	2.9%	2.4%	0.3%	0.0%	0.0%	0.4%	0.6%	0.5%
	Skin	0.0%	0.5%	0.8%	0.8%	0.0%	0.0%	0.0%	0.1%	0.1%	0.1%
	Stomach	0.0%	2.1%	5.3%	2.9%	0.3%	0.5%	1.0%	2.6%	1.9%	0.7%
Mortality	Bladder	0.0%	0.4%	1.7%	1.2%	0.0%	–	–	–	–	–
	Colon and rectum	0.0%	2.5%	7.9%	7.5%	0.8%	–	–	–	–	–
	Esophagus	0.0%	1.2%	2.1%	2.1%	0.0%	–	–	–	–	–
	Female breast	0.0%	1.2%	1.7%	0.4%	0.4%	–	–	–	–	–
	Gallbladder	0.0%	0.8%	3.7%	1.7%	0.8%	–	–	–	–	–
	Kidney	0.0%	0.0%	0.4%	0.8%	0.0%	–	–	–	–	–
	Liver	0.0%	0.8%	5.0%	2.9%	0.4%	–	–	–	–	–
	Lung	0.0%	6.2%	7.5%	3.3%	0.4%	–	–	–	–	–
	Lymphoma	0.0%	0.4%	2.1%	0.8%	0.0%	–	–	–	–	–
	Miscellaneous	0.0%	1.2%	2.1%	0.0%	0.0%	–	–	–	–	–
	Myeloma	0.0%	0.8%	2.1%	0.8%	0.4%	–	–	–	–	–
	Pancreas	0.0%	1.2%	2.9%	2.5%	0.4%	–	–	–	–	–
	Prostate	0.0%	0.0%	2.1%	2.9%	0.8%	–	–	–	–	–
	Stomach	0.0%	1.7%	5.0%	2.9%	0.8%	–	–	–	–	–

Group 1, <50 years; group 2, 50–59 years; group 3, 60–69 years; group 4, 70–79 years; group 5, ≥80 years.

### 3.5 Age- and gender-specific prevalence rates of the patients with malignancy

For *S. japonicum* patients, group 3 (60–69 years) had the most malignancy cases (lung cancer) in men. Group 4 (70–79 years) had the most malignancy cases (colorectal cancer) in women (**Supplementary Table 1**).

For non-*S. japonicum* patients, group 3 (60–69 years) had the most malignancy cases (lung cancer) in men and the most malignancy cases (lung cancer) in women (**Supplementary Table 1**).

### 3.6 Age- and gender-specific mortality of the patients with malignancy

For *S. japonicum* patients, lung cancer caused the highest number of death cases in group 2 (50–59 years) in men. Gallbladder cancer and colorectal cancer caused the highest number of death cases in group 2 (50–59 years) and group 3 (60–69 years) in women, respectively.

### 3.7 Age- and gender-specific 5-year overall survival of the patients with malignancy

**Figure 6:**
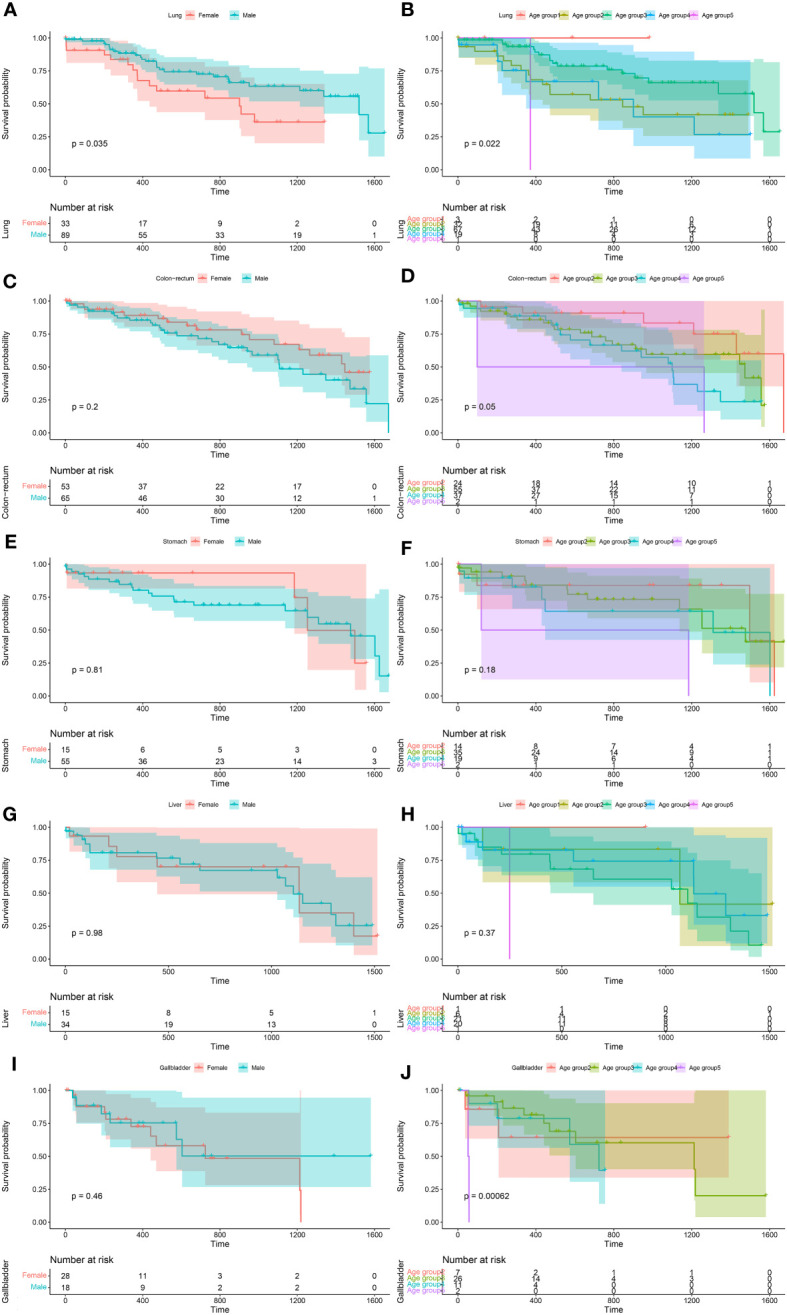
The age- and gender-specific 5-year overall survival of the five leading malignancies of *Schistosoma japonicum* patients. Lung cancer **(A, B)**, colorectal cancer **(C, D)**, stomach cancer **(E, F)**, liver cancer **(G, H)**, and gallbladder cancer **(I, J)**.

For *S. japonicum* patients, their 5-year overall survival of each cancer site is shown in **Supplementary Figure 1**. The age- and gender-specific 5-year overall survival of the patients with five leading malignancies are shown in **Figure 6**.

For *S. japonicum* patients, no significant differences in the survival rates were seen between male and female patients in the cancer sites of the bladder (p = 0.21), colon and rectum (p = 0.20), esophagus (p = 0.97), gallbladder (p = 0.46), kidney (p = 0.52), liver (p = 0.98), lymph nodes (lymphoma) (p = 0.98), blood (myeloma) (p = 0.92), pancreas (p = 0.15), skin (p = 1.00), stomach (p = 0.81), and miscellaneous (p = 0.17). However, higher 5-year overall survival were found in female patients with lung cancer (p = 0.04) (**Supplementary Figure 2**).

For *S. japonicum* patients, no significant differences in the survival rates were seen among the age groups in the cancer sites of the bladder (p = 0.54), esophagus (p = 0.98), kidney (p = 0.26), liver (p = 0.37), lymphoma (p = 0.73), pancreas (p = 0.34), skin (p = 1.00), stomach (p = 0.18), and miscellaneous (p = 0.57). Significant differences in the survival rates were seen among the age groups in the cancer sites of the colon and rectum (p = 0.05), female breast (p = 0.004), lung (p = 0.02), gallbladder (p < 0.001), myeloma (p = 0.04), and prostate (p < 0.001) (**Supplementary Figure 3**).

## 4 Discussion

This study found that lung cancer was the most common malignancy in male *S. japonicum* patients, followed by colorectal cancer. On the contrary, colorectal cancer was found as the most common in female *S. japonicum* patients, followed by lung cancer. However, colorectal cancer was the leading cause of death cases in both genders of *S. japonicum* patients.

The association between *S. japonicum* infection and colorectal cancer has been reported in previous studies (6, 7). A high incidence of colorectal cancer was observed in endemic regions, which was attributed to the high prevalence rate of individuals infected by *S. japonicum* (8). The standardized mortality for colorectal cancer was reported to be significantly higher in female patients who lived in the endemic area for more than 50 years (9). The peak prevalence rate of sporadic colorectal cancer was reported to occur in the 60–80 years of life worldwide (10). However, due to early exposure to *Schistosoma* in childhood, colorectal cancer occurs 6–16 years earlier in patients with schistosomiasis infection than in patients with ordinary colorectal cancer (6, 11). Similar results were shown in this study. The peak prevalence rate of colorectal cancer occurred in the 60–69 years of life in *S. japonicum* patients, while the peak prevalence rate occurred in the 60–69 and 70–79 years of life in non-*S. japonicum* patients. Colorectal cancer was found to be the leading cause of malignancy death in both *S. japonicum* and non-*S. japonicum* patients. The peak mortality occurred in the 60–80 years of life. Previous studies have reported that schistosomiasis would lead to the onset of colon cancer at an age less than 40. The results of this study showed that the onset time of colon cancer for *S. japonicum* patients was approximately 50 years old. This disparity may be a result of the Shanghai colorectal cancer screening program, which was launched in 2012. Precancerous lesions such as colonic polyps could be detected early and treated before developing into colon cancer. However, this study did not include precancerous lesions. Thus, a decreased number of colon cancer cases was observed in younger patients who had been recently infected by *S. japonicum*.

A previous study demonstrated an association between *S. mansoni* and liver cancer, which was probably through the effect of co-infection of hepatitis virus (12). Patients infected by *S. mansoni* have higher rates of co-infection of the hepatitis B virus and hepatitis C virus than the controls (13). Another study analyzed the age distribution among *S. mansoni* patients with liver cancer. Results revealed that patients with co-infection of hepatitis C virus and *S. mansoni* were younger (50–59 years) in age at the time of diagnosis of liver cancer than the patients with hepatitis C virus alone (at the age of 60 years). The combination of *S. mansoni* and hepatitis C virus is frequently associated with more rapid progression to liver cancer (14). A previous study suggested that *S. japonicum* infection may contribute to the disease burden of the liver and finally cause liver cancer (5). In this study, we did not investigate hepatitis B infection among *S. japonicum* patients with liver cancer. However, the results showed that both the peak prevalence rate and peak mortality occurred in the sixth decade of life in *S. japonicum* patients with liver cancer, which was consistent with Shousha’s research (15).

A previous study reported that the clinicopathologic pattern of bladder cancer was different in schistosomiasis and non-schistosomiasis patients. In Western countries, bladder cancer was three times more common in men than in women, with the prevalence rate peaking in the 60–90 years of life. Over 90% of the tumors were transitional cell carcinomas (16). The ratio of bladder cancer incidence (men to women) in countries with *Schistosoma* infection was reported to be within the range of 4:1 to 5.9:1 (17). In this study, the result was consistent with the previous study. A ratio of 4.9 (men to women) was found in the prevalence rate of bladder cancer in *S. japonicum* patients.

Lung cancer was found the most common malignant tumor in both *S. japonicum* and non-*S. japonicum* patients. To the best of our knowledge, there are no clinical reports about the relationship between schistosomiasis and lung cancer. Results of this study showed that the proportion of lung cancer in malignant tumors was 18.6% for *S. japonicum* patients, which was lower than that (30.2%) of non-*S. japonicum* patients. This result indicated that lung cancer may not be related to parasite infection. However, controversy remains in basic studies on the potential relationship between *S. japonicum* and lung neoplasm. Chen et al. reported that *S. japonicum* infection may contribute to pulmonary granulomatous inflammation (18). Lin et al. reported that infection of *S. japonicum* was likely to enhance the proliferation and migration of human breast cancer cells rather than lung A549 cancer cells (19).

Previous studies have reported that hepatitis B, *Schistosoma*, and multiple pregnancies may affect the cancer process of the gallbladder (20). However, the mechanism was still unclear. One case report presented a case of co-occurrence of *S. mansoni* and stomach cancer. The pathological mechanism may lead to pseudo-tumor formation due to excessive hyper-plastic neoformation and anomalous response to *Schistosoma* (21). Other cancer co-occurrences with schistosomiasis such as prostate adenocarcinoma associated with *S. haematobium* infection were previously reported (22). However, the specific mechanism was not clear either.

This study had some limitations. First, the present retrospective study included limited samples of single-center data. Further study should be carried out in prospective studies in multi-centers with larger sample sizes. Second, with the limited clinical information, the relationship between various malignancies other than digestive system tumors and *S. japonicum* could not be clarified. Third, personal lifestyle and habits were not investigated in the current study, which may be related to the occurrence of malignancies such as smoking or the presence of carcinogens in the work environment. Furthermore, co-infection with other pathogens may exist in *S. japonicum* patients, which may lead to potential bias. Thus, more comprehensive research needs to be further performed.

## 5 Conclusions

In conclusion, *S. japonicum* is attributed to a high prevalence rate and mortality in digestive system tumors, including colorectal cancer, stomach cancer, liver cancer, and gallbladder cancer.

## Data availability statement

The raw data supporting the conclusions of this article will be made available by the authors, without undue reservation.

## Ethics statement

The studies involving humans were approved by Ethics Committee of Jinshan Hospital, Fudan University. The studies were conducted in accordance with the local legislation and institutional requirements. The ethics committee/institutional review board waived the requirement of written informed consent for participation from the participants or the participants’ legal guardians/next of kin because Informed consent was waived. Patients’ confidentiality was protected. All the data were addressed in anonymous. The personal information was appropriately de-identified.

## Author contributions

X-FL: Data curation, Formal Analysis, Investigation, Writing – original draft. SJ: Data curation, Writing – review & editing. K-YW: Data curation, Writing – review & editing. YL: Conceptualization, Data curation, Formal Analysis, Funding acquisition, Investigation, Writing – original draft, Writing – review & editing. J-WQ: Funding acquisition, Writing – review & editing.
