# Supplementary material for: The prevalence rate, mortality, and 5-year overall survival of Schistosoma japonicum patients with human malignancy
Source: Front Oncol. 2023 Dec 6;13:1288197. doi: 10.3389/fonc.2023.1288197 (PMC10731309; doi:10.3389/fonc.2023.1288197)
Supplement: Supplementary file 1 [file DataSheet_1.docx]

Supplementary Material

# Supplementary Figures and Tables


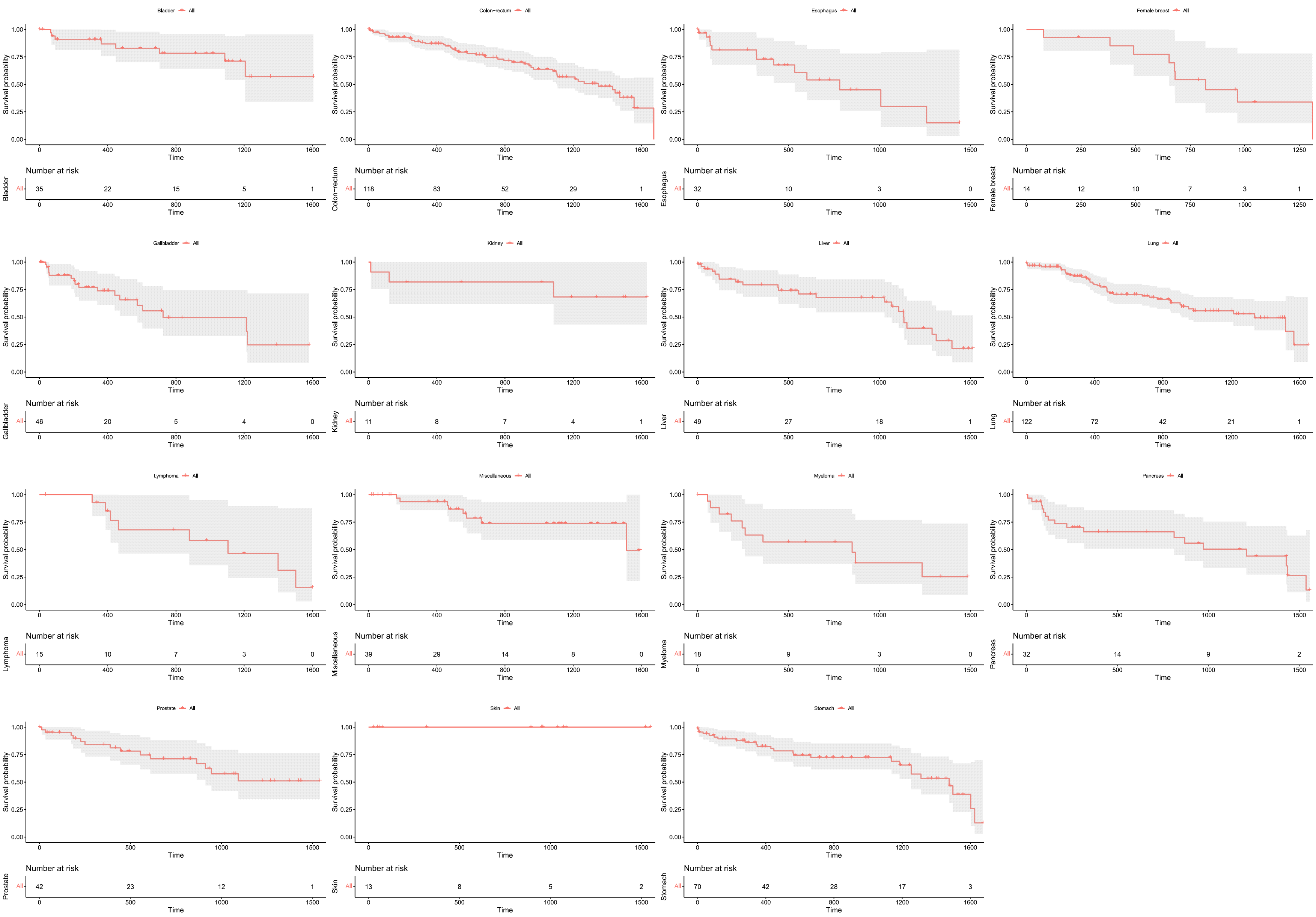


**Supplementary Figure 1. The five-year overall survival of each cancer in *S. japonicum* patients.**


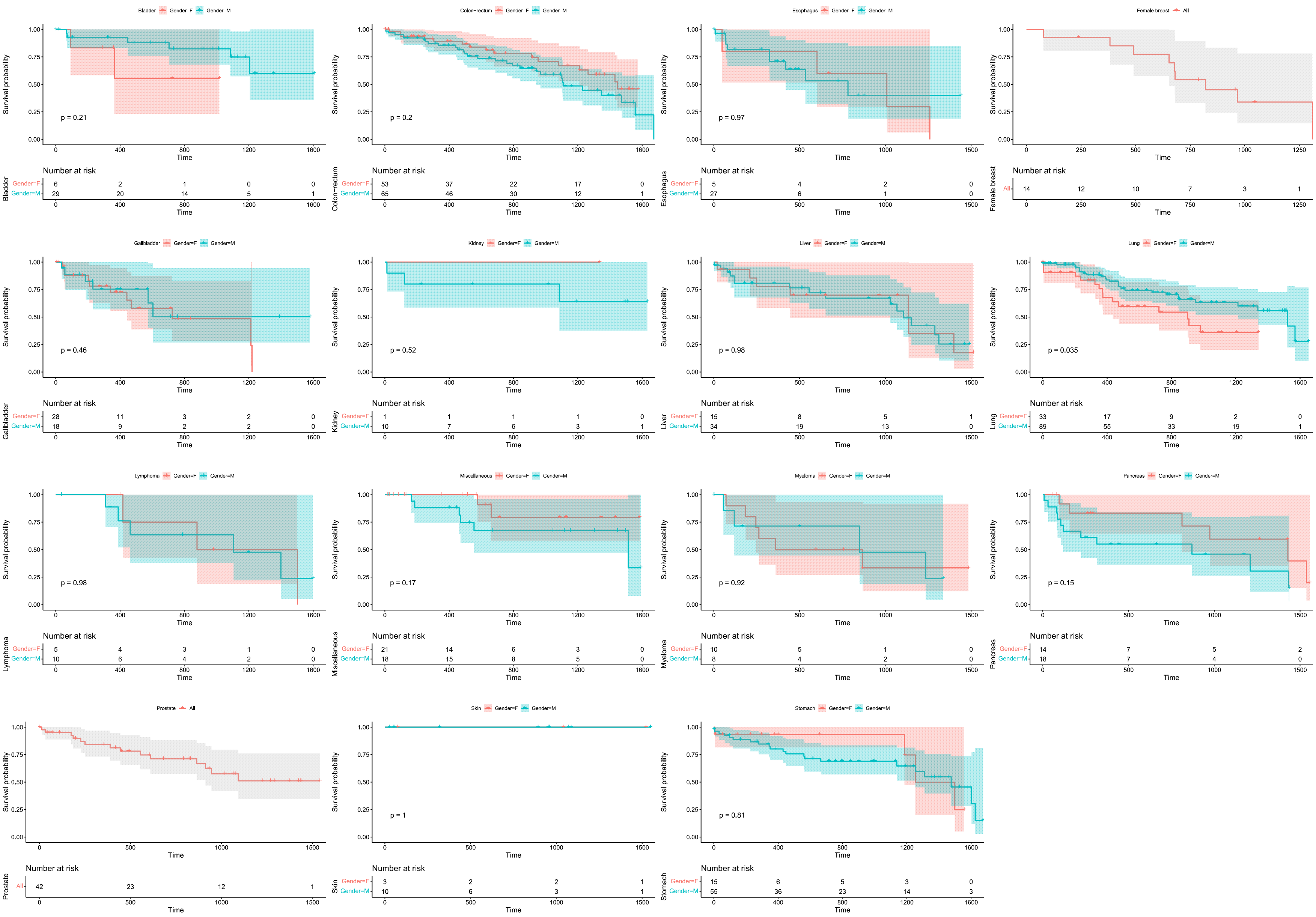


**Supplementary Figure 2. The gender-specific five-year overall survival of each cancer in *S. japonicum* patients.**


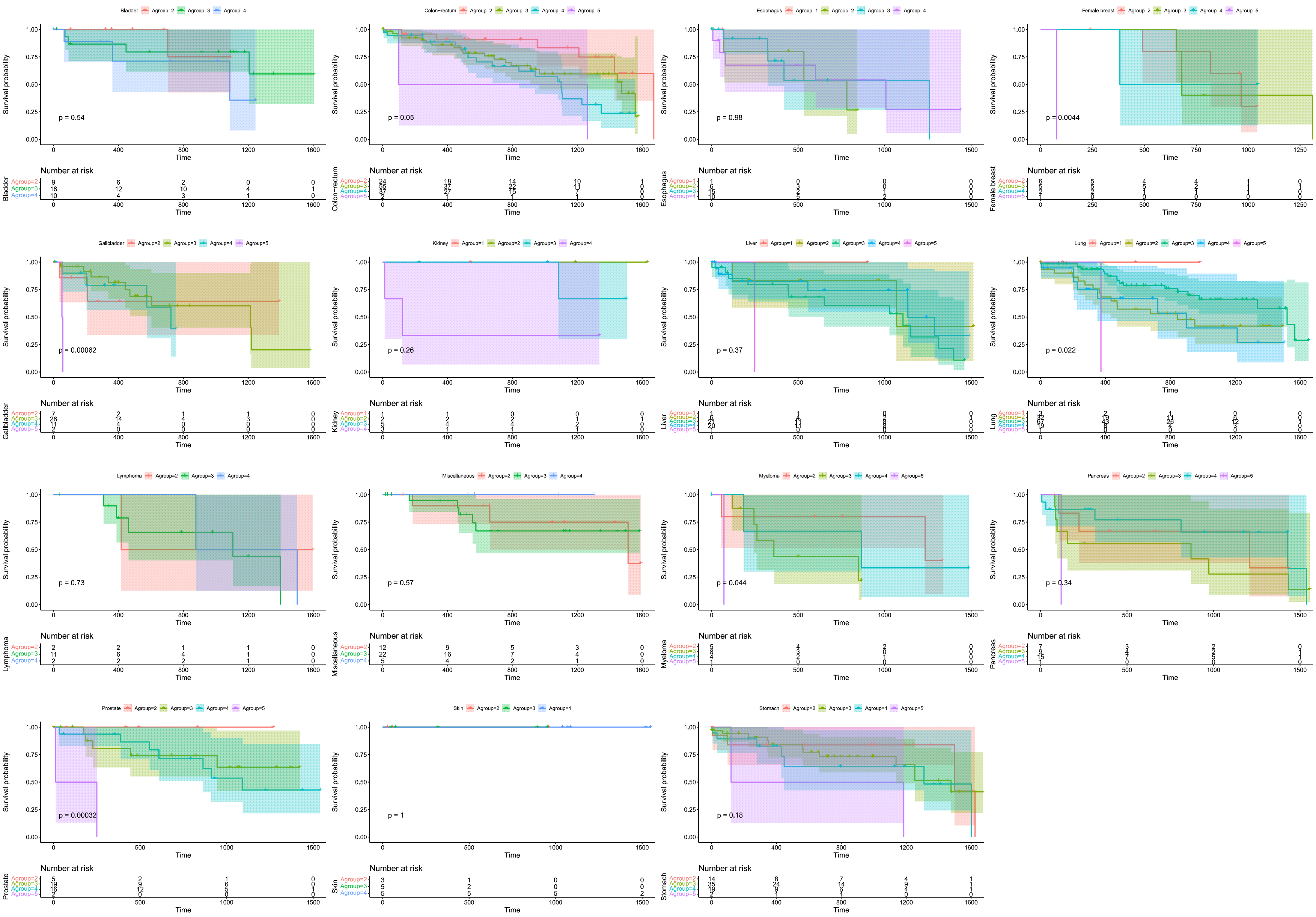


**Supplementary Figure 3. The age-specific five-year overall survival of each cancer in *S. japonicum* patients.**

**Supplementary Table 1.** **The prevalence by gender and age groups in *S. japonicum* and non*-S. japonicum* patients.**

| Gender | Cancer site | Schistosomiasis patients | | | | | non-Schistosomiasis patients | | | | |
| --- | --- | --- | --- | --- | --- | --- | --- | --- | --- | --- | --- |
|  |  | Group 1 | Group 2 | Group 3 | Group 4 | Group 5 | Group 1 | Group 2 | Group 3 | Group 4 | Group 5 |
| Male | Bladder | 0.00% | 1.22% | 2.13% | 1.07% | 0.00% | 0.20% | 0.30% | 1.00% | 0.83% | 0.31% |
|  | Colon-rectum | 0.00% | 1.68% | 5.64% | 2.59% | 0.00% | 1.31% | 1.72% | 2.84% | 2.55% | 1.24% |
|  | Esophagus | 0.15% | 0.91% | 1.98% | 1.07% | 0.00% | 0.04% | 0.52% | 1.99% | 1.39% | 0.48% |
|  | Gallbladder | 0.00% | 0.30% | 1.98% | 0.30% | 0.15% | 0.18% | 0.44% | 0.78% | 0.63% | 0.22% |
|  | Kidney | 0.15% | 0.30% | 0.76% | 0.30% | 0.00% | 0.76% | 0.90% | 1.00% | 0.59% | 0.26% |
|  | Liver | 0.15% | 0.61% | 2.29% | 2.13% | 0.00% | 0.68% | 0.89% | 0.90% | 0.57% | 0.37% |
|  | Lung | 0.46% | 3.66% | 7.93% | 1.52% | 0.00% | 1.02% | 3.64% | 8.66% | 6.41% | 1.90% |
|  | Lymphoma | 0.00% | 0.15% | 1.37% | 0.00% | 0.00% | 0.22% | 0.26% | 0.42% | 0.18% | 0.09% |
|  | Miscellaneous | 0.00% | 0.76% | 1.68% | 0.30% | 0.00% | 0.90% | 1.16% | 1.35% | 0.81% | 0.33% |
|  | Myeloma | 0.00% | 0.46% | 0.61% | 0.15% | 0.00% | 0.04% | 0.04% | 0.11% | 0.13% | 0.02% |
|  | Pancreas | 0.00% | 0.91% | 0.76% | 0.91% | 0.15% | 0.15% | 0.46% | 0.89% | 0.44% | 0.24% |
|  | Prostate | 0.00% | 0.76% | 2.90% | 2.44% | 0.30% | 0.04% | 0.02% | 0.35% | 0.57% | 0.46% |
|  | Skin | 0.00% | 0.46% | 0.61% | 0.46% | 0.00% | 0.02% | 0.02% | 0.04% | 0.07% | 0.04% |
|  | Stomach | 0.00% | 1.52% | 4.57% | 2.13% | 0.15% | 0.26% | 0.63% | 1.94% | 1.37% | 0.33% |
| Female | Bladder | 0.00% | 0.15% | 0.30% | 0.46% | 0.00% | 0.00% | 0.09% | 0.13% | 0.20% | 0.09% |
|  | Colon-rectum | 0.00% | 1.98% | 2.74% | 3.05% | 0.30% | 0.00% | 1.35% | 1.81% | 1.88% | 0.87% |
|  | Esophagus | 0.00% | 0.00% | 0.30% | 0.46% | 0.00% | 0.00% | 0.04% | 0.06% | 0.31% | 0.24% |
|  | Female breast | 0.00% | 0.91% | 0.76% | 0.30% | 0.15% | 0.00% | 1.29% | 1.31% | 0.42% | 0.07% |
|  | Gallbladder | 0.00% | 0.76% | 1.98% | 1.37% | 0.15% | 0.00% | 0.31% | 0.59% | 0.41% | 0.42% |
|  | Kidney | 0.00% | 0.00% | 0.00% | 0.15% | 0.00% | 0.00% | 0.52% | 0.52% | 0.41% | 0.24% |
|  | Liver | 0.00% | 0.30% | 0.91% | 0.91% | 0.15% | 0.00% | 0.20% | 0.30% | 0.42% | 0.30% |
|  | Lung | 0.00% | 1.22% | 2.29% | 1.37% | 0.15% | 0.00% | 2.40% | 3.47% | 2.90% | 1.05% |
|  | Lymphoma | 0.00% | 0.15% | 0.30% | 0.30% | 0.00% | 0.00% | 0.17% | 0.22% | 0.17% | 0.07% |
|  | Miscellaneous | 0.00% | 1.07% | 1.68% | 0.46% | 0.00% | 0.00% | 1.57% | 0.96% | 0.81% | 0.15% |
|  | Myeloma | 0.00% | 0.30% | 0.61% | 0.46% | 0.15% | 0.00% | 0.02% | 0.07% | 0.13% | 0.02% |
|  | Pancreas | 0.00% | 0.15% | 0.61% | 1.37% | 0.00% | 0.00% | 0.22% | 0.54% | 0.37% | 0.31% |
|  | Skin | 0.00% | 0.00% | 0.15% | 0.30% | 0.00% | 0.00% | 0.00% | 0.04% | 0.06% | 0.06% |
|  | Stomach | 0.00% | 0.61% | 0.76% | 0.76% | 0.15% | 0.00% | 0.35% | 0.66% | 0.52% | 0.35% |

group 1: < 50 y; group 2: 50-59 y; group 3: 60-69 y; group 4: 70-79 y; group 5: ≥ 80 y

**Supplementary Table 2. The malignancy mortality by gender and age groups of *S. japonicum* patients.**

| Gender | Cancer site | Mortality | | | | |
| --- | --- | --- | --- | --- | --- | --- |
|  |  | Group 1 | Group 2 | Group 3 | Group 4 | Group 5 |
| Male | Bladder | 0.41% | 1.24% | 0.83% | 0.00% | 0.00% |
|  | Colon-rectum | 1.24% | 5.81% | 4.98% | 0.00% | 0.00% |
|  | Esophagus | 1.24% | 1.66% | 0.83% | 0.00% | 0.00% |
|  | Gallbladder | 0.41% | 1.24% | 0.41% | 0.41% | 0.00% |
|  | Kidney | 0.00% | 0.41% | 0.83% | 0.00% | 0.00% |
|  | Liver | 0.83% | 3.73% | 1.66% | 0.00% | 0.00% |
|  | Lung | 3.73% | 6.22% | 1.24% | 0.00% | 0.00% |
|  | Lymphoma | 0.00% | 2.07% | 0.00% | 0.00% | 0.00% |
|  | Miscellaneous | 0.83% | 1.66% | 0.00% | 0.00% | 0.00% |
|  | Myeloma | 0.83% | 0.83% | 0.00% | 0.00% | 0.00% |
|  | Pancreas | 1.24% | 1.66% | 1.24% | 0.41% | 0.00% |
|  | Prostate | 0.00% | 2.07% | 2.90% | 0.83% | 0.00% |
|  | Skin | 0.00% | 0.00% | 0.00% | 0.00% | 0.00% |
|  | Stomach | 1.24% | 4.56% | 2.49% | 0.41% | 0.00% |
| Female | Bladder | 0.00% | 0.41% | 0.41% | 0.00% | 0.00% |
|  | Colon-rectum | 1.24% | 2.07% | 2.49% | 0.83% | 0.00% |
|  | Esophagus | 0.00% | 0.41% | 1.24% | 0.00% | 0.00% |
|  | Female breast | 1.24% | 1.66% | 0.41% | 0.41% | 0.00% |
|  | Gallbladder | 0.41% | 2.49% | 1.24% | 0.41% | 0.00% |
|  | Kidney | 0.00% | 0.00% | 0.00% | 0.00% | 0.00% |
|  | Liver | 0.00% | 1.24% | 1.24% | 0.41% | 0.00% |
|  | Lung | 2.49% | 1.24% | 2.07% | 0.41% | 0.00% |
|  | Lymphoma | 0.41% | 0.00% | 0.83% | 0.00% | 0.00% |
|  | Miscellaneous | 0.41% | 0.41% | 0.00% | 0.00% | 0.00% |
|  | Myeloma | 0.00% | 1.24% | 0.83% | 0.41% | 0.00% |
|  | Pancreas | 0.00% | 1.24% | 1.24% | 0.00% | 0.00% |
|  | Skin | 0.00% | 0.00% | 0.00% | 0.00% | 0.00% |
|  | Stomach | 0.41% | 0.41% | 0.41% | 0.41% | 0.00% |

group 1: < 50 y; group 2: 50-59 y; group 3: 60-69 y; group 4: 70-79 y; group 5: ≥ 80 y
